# Treatment fidelity in the Tinnitus Retraining Therapy Trial

**DOI:** 10.1186/s13063-020-04530-9

**Published:** 2020-07-23

**Authors:** Roberta W. Scherer, Sue Ann Erdman, Susan Gold, Craig Formby, for the TRTT Research Group

**Affiliations:** 1 Center for Clinical Trials and Evidence Synthesis, Department of Epidemiology, Johns Hopkins Bloomberg School of Public Health, Baltimore, MD USA; 2 Audiologic Rehabilitation Consulting Services, Jensen Beach, FL USA; 3 Tinnitus and Hyperacusis Center of Maryland, Department of Otolaryngology, University of Maryland Medical Center, Baltimore, MD USA; 4 Department of Communicative Disorders, University of Alabama, Tuscaloosa, AL USA

**Keywords:** Treatment fidelity, Protocol adherence, Tinnitus, Randomized trial

## Abstract

**Background:**

Treatment fidelity, defined as ensuring that the recipient receives the intended intervention, is a critical component for accurate estimation of treatment efficacy. Ensuring fidelity and protocol adherence in behavioral trials requires careful planning during the design phase and implementation during the trial. The Tinnitus Retraining Therapy Trial (TRTT) randomized individuals with severe tinnitus to tinnitus retraining therapy (TRT, comprised of tinnitus-specific educational counseling (TC) and sound therapy (ST) using conventional sound generators (SGs)); Partial TRT (TC and placebo SGs); or standard of care (SOC), using a patient-centered care approach. Study audiologists administered both types of counseling in the TRTT, creating a challenge for managing protocol adherence.

**Methods:**

We developed methods to enhance treatment fidelity including training, competency assessment, scripts, visual aids, and fidelity monitoring. Protocol monitors identified critical topics and content to be addressed for each type of counseling session, prepared corresponding scripts, and developed training aids and treatment-specific checklists covering those topics. Study audiologists’ competency assessment required submission and review by the protocol monitors of an audiotape of one TC and one SOC counseling session. Treatment-specific aids included scripts, a 3-D model of the ear, handouts, and for TC, an illustrated flip-chart with talking points that followed the scripted content. During the trial, audiologists completed treatment-specific checklists during each counseling session, indicating topics covered/discussed and submitted audiotapes of counseling sessions. Protocol monitors reviewed audiotapes using corresponding treatment-specific checklists. Results for individual checklist items were tabulated and proportions calculated.

**Results:**

Twenty-five audiologists were certified for TC and/or SOC counseling and 24 completed at least one counseling session. Adherence to each of 33 critical items on the TC checklist as assessed by the protocol monitor ranged from 70 to 100% across 37 counseling sessions (median 97%), with no difference between adherence for TRT (median, 97%) and partial TRT (median, 100%). Adherence to each of 44 critical items on the SOC checklist across 30 SOC counseling sessions ranged from 42 to 100% (median, 87.5%).

**Conclusion:**

The TRTT used multiple methods to address treatment fidelity. The close adherence to each treatment type was critical for evaluating the efficacy of the study interventions in this randomized trial.

**Trial registration:**

clinicaltrials.gov NCT01177137. Registered on 5 August 2010.

## Background

Adherence or fidelity is a critical component of randomized trials. Importantly, treatment fidelity impacts the ability of investigators to estimate accurately the treatment effect; poor adherence to the treatment protocol may result in enhancement of either type I or type II errors. When the intervention is not a drug, but a behavioral intervention, treatment fidelity allows for the identification of the critical elements of the experimental and comparator interventions for replication in future studies or in practice. Ultimately, poor adherence can lead to inaccurate translation of trial findings to clinical practice.

In medication trials, adherence monitoring may involve counting unused tablets or pills or measurement of a biochemical marker. However, when the intervention involves a counseling or behavioral component, measures to assess adherence are less well-developed. Treatment fidelity for behavioral interventions is defined as ensuring that the recipient receives the intended intervention. Measures to enhance treatment fidelity in trials aim to confirm that individuals take the medication or receive the type of counseling or other intervention assigned at randomization. These measures also aim to prevent errors due to contamination of the treatment. Typically, treatment fidelity involves a number of steps, as recommended by the National Institutes of Health (NIH) Behavior Change Consortium and described by Bellg et al. [1]. These steps include:

- **Design**: strategies to enhance fidelity in delivery of treatment (e.g., prompts, similar contacts, sufficient persons to deliver treatment). Design addresses the question “What steps can be taken to make sure that the participant receives the intended intervention?”
- **Training**: training of providers who will deliver the intervention (standardization, strategies to minimize drift). Training addresses the question “Are the providers adequately trained to deliver the intervention?”
- **Delivery**: processes to monitor and improve delivery of the intervention (e.g., scripted intervention). Delivery addresses the question “Do the providers deliver the intervention as intended?”
- **Receipt**: processes to monitor and improve ability of participants to understand and implement the intervention (e.g., tests, interactive interviews). Receipt addresses the question “Do the participants understand the intervention and are they able to use it?”
- **Enactment**: processes to monitor and improve ability of participants to implement intervention in real-life settings (questionnaires, interviews). Enactment addresses the question “Are the participants implementing the intervention following the intervention?”

Thoughtful pre-planning is required to implement these adherence steps to achieve consistency and accuracy for most behavioral interventions. The details of these steps often varies across trials. For example, some recent reports described fidelity steps for a variety of interventions, including music therapy for people with dementia [2], best practices for implementing mindfulness-based interventions [3], or motivational interviewing [4]. Trials that report on measures taken to enhance treatment fidelity tend to emphasize delivery, rather than participant engagement in treatment [5]. Although researchers recognize the importance of implementing measures for treatment fidelity, few have received adequate training in fidelity measures or include descriptions of these measures in trial reports [6]. Excellent resources for planning include the model developed by the NIH Behavior Change Consortium, as described by Bellg et al. [1] and Borrelli [6] and implemented by Kechter et al. [3] and Robb et al. [7]. There are also various articles describing methods used for fidelity monitoring either generally [8] or across many areas of behavioral interventions as described above.

Tinnitus, the perception of sound in the absence of a corresponding external stimulus for which there is no apparent cause is a debilitating condition for some individuals. Various options for management exist, including tinnitus retraining therapy (TRT). TRT involves non-psychiatric tinnitus-specific educational counseling (TC) and low-level sound therapy (ST), typically achieved using sound generators (SGs) and/or enriched environmental sound, to habituate the individual’s associated negative emotional reactions (e.g., annoyance or anxiety) and perception (awareness) of the tinnitus, and ultimately its impact on the patient’s life [9]. The overall goal of TC is to educate the patient about his or her condition, which is achieved through didactic education. We compared the efficacy of TRT with the standard of care (SOC) in the Tinnitus Retraining Therapy Trial (TRTT). The TRTT, a multi-center trial conducted at US military medical sites, enrolled individuals with severe tinnitus to the trial. The TRTT also evaluated the necessity for the use of SGs by including a third study arm, the Partial TRT group, which used TC and short-acting placebo SGs as a control for ST. The SOC group received counseling that used an interactive patient-centered care approach focusing on assisting the study patient to become actively engaged in his or her symptom relief [10]. All three study groups were encouraged to use environmental sound at all times. No difference was found between treatment groups on numerous measures of tinnitus-specific health-related quality of life [11], so it is critical to emphasize that these were distinct interventions. Table 1 summarizes the main differences between the two types of counseling, TC and SOC. All three treatment groups involved one-on-one counseling, with the TRT and Partial TRT groups receiving TC counseling. Study audiologists administered both types of counseling in the TRTT primarily to avoid a counselor effect, minimize the required number of audiologists, and allow all audiologists to receive training in TRT and the patient-centered approach for SOC. Blinding was maintained by having one audiologist provide the intervention (counseling) and the second measure all audiologic outcomes for an individual participant. To avoid cross-contamination, it was necessary to develop a protocol to enhance adherence to each management strategy. In this report, we describe how we followed the steps recommended by the NIH Behavior Change Consortium and described by Bellg et al. [1] to achieve treatment fidelity in the TRTT.

**Table 1:** Comparison of TRT counseling (TC) and SOC counseling

TRT counseling	Standard of care counseling
Based on Jastreboff’s neurophysiological model	Based on existing practice and American Speech-Language-Hearing Association (ASHA) guidelines
Theory-driven	Patient-driven
Directive	Facilitative
Didactic/top-down	Interactive/horizontal

## Methods

### TRTT trial design

The TRTT protocol has been previously described [9]. Briefly, we assessed the efficacy of TRT and its component parts, TC and ST, compared with SOC in habituating the perceived magnitude, perception, and negative emotional reactions to tinnitus. We conducted the trial in US Air Force, Navy, and integrated Department of Defense Medical Centers and enrolled active-duty and retired military personnel and their dependents. Eligibility criteria included subjective distressing tinnitus of at least 1 year’s duration with no known medical etiology; functionally adequate hearing sensitivity; no treatment for tinnitus within the past year; and a score of 40 or more on the Tinnitus Questionnaire (TQ) [12], indicating a moderately severe impact of tinnitus on quality of life. Assignments using a computer-generated random schedule with random blocking and stratification by clinical center were implemented via the TRTT website. The sample size of 228 was based on a 10-point difference in TQ scores between baseline and 18 months’ follow-up for the primary comparison (between TRT and SOC) and 7 points for comparisons of ST and TC, *α* of 0.05, 80% power, standard deviation of 12.5, and a two-sided test and 10% attrition. The primary outcome, mean change in TQ score from baseline to follow-up, was assessed longitudinally at 3, 6, 12, and 18 months’ follow-up using intention-to-treat longitudinal data analyses. End-of-treatment and longitudinal changes in scores on the sub-scales of the TQ were secondary outcomes; other outcomes include the total and subscale scores of the Tinnitus Functional Index [13], Tinnitus Handicap Inventory [14], and change on a 10-point Visual Analogue Scale from the TRT Interview Form [15].

The TRTT received ethical approval from institutional review boards at the University of Alabama, the Johns Hopkins Bloomberg School of Public Health, and participating clinical sites. No study participant was enrolled in the study before informed consent was obtained and documented by a signed informed consent statement.

### Adherence

We summarize the steps we used to match the NIH Behavior Change Consortium model [1] in Table 2 and describe them in detail below.

**Table 2:** Incorporation of TRTT counseling protocol into the NIH Behavior Change Consortium model [1] of treatment fidelity

Goal	TRTT strategies
**Design**	
Ensure same treatment dose within different settings	Development of a standard protocol for counseling to administer at military hospitals across the country by audiologists with differences in previous experience in treating patients with tinnitus.
Ensure equivalent dose across conditions	Counseling conducted at treatment visits conducted within 2 months following randomization and a follow-up treatment visit 1 month later.
Plan for implementation setbacks	Standard certification requirements for replacement of study audiologists during the trial.
**Training providers**	
Standardize training	Completion of training by reading relevant sections of the TRTT Manual of Procedures and (1) attendance at a two-day regional training session, (2) attendance at a webinar, or (3) viewing a videotape of a counseling session.
Ensure provider skill acquisition	Competency assessed through submission of a “dummy” audiotape of each type of counseling session for review by the protocol monitors
Minimize “drift” in provider skills	Submission of randomly selected audiotapes during the trial for review by protocol monitors.
Accommodate provider differences	Scripts with suggested wording for both types of counseling, but with allowances for individual styles.
**Monitoring and improving delivery of treatment**	
Control for provider differences	Checklists used to ensure adherence to critical components.
Reduce difference within treatment	Separate scripts and visual aids to be used during counseling sessions including an instructive flip chart and 3-D ear models.
Ensure adherence to treatment protocol	Submission of audiotapes and checklists of first two of each type of counseling session for review by protocol monitors; submission of randomly selected audiotapes and checklists after sessions initially reviewed.
Minimize contamination between conditions	Separate checklists and scripts for each type of counseling session.
**Improving receipt of treatment**	
Ensure participant comprehension	Interactive sessions with opportunity for questioning both by participant and family.
Ensure participant ability to use cognitive skills	Handouts that summarized concepts covered during counseling that participants could take home.
Ensure participant ability to perform behavioral skills	Practice during the counseling session and handouts that included symptom management strategies to be practiced following the session.
**Enactment of treatment skills**	
Ensure participant use of cognitive and behavioral skills	Assessment of impact of tinnitus through administration of tinnitus-specific health-related quality of life instruments

### Design of steps for adherence

Adherence design involves “strategies to enhance fidelity in delivery of treatment (e.g., prompts, similar contacts, sufficient persons to deliver treatment)”. Steps we implemented to address adherence design included engagement of experts to develop standardized protocols, development of checklists, and decisions regarding the number of audiologists and how they were expected to administer treatment within the trial. The TRTT engaged two audiologic experts, one each in TC [9] and SOC [10], to develop the protocols for counseling in the TRTT and serve as protocol monitors. The protocol monitors developed the protocol, trained audiologists, and monitored the quality of the administration of counseling sessions. The first step was development of a standard protocol for counseling, keeping in mind that the protocol would be administered at military hospitals across the country and by a number of audiologists with differences in previous experience in treating patients with tinnitus. To achieve consistency across clinical sites, each protocol monitor first identified critical topics for each type of counseling session. This effort included survey of the existing care for tinnitus patients at each site preliminary to establishing the TC protocol [10]. These critical steps were incorporated into a written protocol that became a chapter within the TRTT Manual of Procedures. A checklist was prepared corresponding to the critical items for audiologists to use during counseling sessions. This checklist then provided an outline for development of the detailed script prepared by each protocol monitor. Corresponding talking points to be used as prompts to help the audiologist present pertinent information specific to the type of counseling being administered, either TC or SOC, were also included in the script. Scripts were not meant to be read verbatim, but served as aids to direct and prompt treating audiologists through the respective counseling sessions. Allowances were made within the scripts for personal counselor style or variation, keeping critical elements intact. These protocols, described in detail elsewhere [9, 10], were considered crucial for the successful standardized management of tinnitus within each arm of the trial across the six participating military clinical centers in the TRTT.

### Training

Training involves “training of providers who will deliver the intervention (standardization, strategies to minimize drift)”. Audiologists for the TRTT were required to have completed at least 1 year of clinical experience (post-clinical fellowship year) as an audiologist. No audiologist was permitted to administer either counseling intervention within the trial before completing training and certification specific to the TRTT and to the TC and/or SOC counseling administration. (Requirements for certification are included in supplemental Table 1). Training for most audiologists involved attendance at 2-day regional training sessions. Each in-person TC training session included a description of the theoretical basis, the neurophysiological model, and important concepts relevant to TRT. Implementation of effective counseling involved a discussion on retraining the neuronal circuitry, TRT categories, and analogies to use to facilitate understanding of difficult topics. Procedures for fitting and accurately setting sound levels for the SGs were also described in detail. Face-to-face training was supplemented with videotapes showing the TRT protocol monitor conducting a counseling session. Additionally, the principles of TRT and the TRT protocol, as described by Jastreboff and Hazell [16] and outlined in the TRTT Manual of Procedures, were reviewed by each study audiologist. Training for SOC counseling covered the theoretical basis of a patient-oriented approach, including concepts of self-efficacy and shared decision-making [10]. The SOC protocol monitor covered possible treatments for bothersome symptoms, including those for sleep, stress management, and concentration. Training for SOC counseling involved role-playing to facilitate methods to elicit the patient’s “story” and methods to emphasize empathy for the patient’s condition, an important aspect of the patient-centered approach. A videotaped counseling session by the protocol monitor supplemented the face-to-face SOC training exercises. Audiologists not able to attend a face-to-face training meeting attended an interactive webinar that emulated the training session. Audiologists not able to attend either an in-person training session or a webinar were able to view videotapes prepared during regional training sessions and videotapes of the protocol monitor conducting counseling sessions, along with reading relevant chapters in the TRTT Manual of Procedures.

Audiologists were encouraged to practice counseling sessions with “dummy” patients. When each counselor felt adequately prepared, he or she submitted a voice recording of one TC and one SOC counseling session, each conducted with a non-study individual. Protocol monitors reviewed the recordings and scored them as acceptable or not acceptable based in part on coverage of the counseling content per the checklist for either TC or SOC, i.e., whether the audiologist demonstrated competency in administering the intervention. Protocol monitors reviewed and certified audiologists with acceptable audiotapes to treat TRTT study patients. For unacceptable audiotapes, protocol monitors itemized issues of concerns and sent suggestions for remediation to the audiologist by email or discussed them with the audiologist during a telephone conversation. Some audiotapes were provisionally accepted, requiring a telephone conversation with the protocol monitor to review a minor aspect of the counseling session that needed improvement. Audiologists with unacceptable tapes could then submit additional audiotapes to demonstrate competence in administering the specified type of counseling.

### Delivery

One of the issues in having the same counselor deliver both types of counseling sessions is the real possibility of overlap or bleeding from the concepts described in one type of counseling delivery to the other. To circumvent this possibility, we developed a number of delivery aids, including scripts with corresponding talking points, handouts, flip charts and other visual aids, and checklists. These addressed the issue described by Bellg et al. as *delivery*, including processes to monitor and improve delivery of the intervention [1]. Scripts and emphasis on delivery aids also enhanced standardization across multiple clinical sites.

To begin development of the protocol for each type of counseling, protocol monitors began by identifying critical topics for each type of counseling session. They then incorporated these topics into scripts and used them to develop checklists covering those topics, keeping in mind the site survey results describing the typical care at the military sites [10]. Because some topics were critical for both TC and SOC counseling sessions (e.g., how we hear), it was important to ensure that the scripts and associated talking points were linked to the visual aids developed for use for each type of counseling. In addition, the scripts ensured the amount of detail and emphasis used in each type of counseling session. For example, audiologists used a model of the ear to explain the anatomy and physiology of hearing in both types of counseling sessions. Although both sessions advised tailoring the facts to match the participants’ level of understanding, the amount of anatomic and physiologic detail provided in the TC counseling session was much greater than that in the SOC session in order to describe fully the neurological basis of Jastreboff’s model. The scripts also provided information that could be used by the counselors to facilitate counseling delivery. For example, the TC script provided analogies that the audiologist could use to explain the difference between a stimulus that was perceived as threatening and one that was not, or an example of how background environment impacts the perception of a stimulus. In contrast, the SOC script provided participants with methods that they could use for stress reduction or shifting attention away from the tinnitus.

Each audiologist also was provided with a flip chart that was used only during a TC counseling session because of the greater emphasis on anatomic and physiologic concepts. On the side of the flipchart viewed by the participant was an illustration, while the opposite side (viewed by the audiologist) described the talking points related to that illustration. The TC presentation started with a diagram of the ear, including the gross anatomy of the components of the ear. The audiologist simultaneously used the first illustration in the flip chart and the three-dimensional model of the ear to describe the anatomy and physiology of the outer, middle, and inner ear and to discuss possible sites of any associated hearing loss and origins of tinnitus. Instructions to the audiologist were to spend enough time on this section to educate a patient about the inner ear and relevant neural pathways so that the presentation of the Jastreboff neurophysiological model of tinnitus would be a logical next step [17]. Altogether, the TC flip chart included 14 illustrations. These charts could also be loaded onto a laptop depending on the preference and experience of the audiologist. The counseling session for the SOC used the 3-dimensional ear model and provided a simplified explanation of the anatomy and physiology of hearing, elaborated upon based on the study participant’s understanding and questions. Other SOC topics were linked to specific handouts, including facts related to “what tinnitus is and isn’t”, environmental sound options, tips on eliminating stress and sleep problems, and ways to minimize the impact of tinnitus on focus and concentration. The SOC’s patient-centered approach was specifically intended to address the issues raised by each individual participant; emphasis was given to those specific problem areas with relevant hand-outs provided to the patient.

It should be emphasized here that the material provided for both the TRT counseling and the SOC counseling sessions allowed reasonable flexibility and that each session was expected to be tailored for the individual participant. The theoretical concepts on which TRT is based were requisite aspects of the TC; an empathic response from the study audiologist and shared decision making regarding treatment goals and options were considered essential in the SOC protocol. In both approaches, audiologists were encouraged to listen to and build a relationship with the participant.

### Monitoring

The other aspect of delivery involved monitoring. We incorporated two approaches for monitoring. The protocol monitors developed separate checklists for TC and SOC counseling sessions. Each comprised a list of topics that were essential to cover during the counseling session. The SOC checklist also included an option to indicate that a specific symptom (e.g., lack of concentration) was not a problem for an individual and, while asked, was not covered in depth. Checklists were completed either during the session or immediately following the session and submitted to the protocol monitors for subsequent review.

In addition to the checklist, counseling sessions were audiotaped, provided that the study patient gave permission. Audiologists submitted their recordings of the first two study counseling sessions of each type. Protocol monitors reviewed the audiotape by completing a separate identical checklist to verify topics covered, checking whether topics were adequately covered during the counseling session. If no deficiencies were noted, then subsequent recordings were randomly selected for review. Whenever deficiencies were noted, the protocol monitor communicated with the audiologist in writing or by phone to discuss the deficiency, and the next two counseling sessions were submitted for review. Continued non-adherence would have resulted in de-certification; however, no audiologists were de-certified during the trial.

### Receipt and enactment

In the NIH Behavior Change Consortium model for treatment fidelity, receipt of the intervention encompasses processes to monitor and improve the ability of participants to understand and implement the intervention [1]. To facilitate understanding of the ideas covered in the counseling session, both TC and SOC counselors prepared handouts that participants could take home. The TC counselor prepared a handout that summarized the TC counseling concepts for all study patients. The SOC counselor prepared a number of handouts that were distributed to study participants based on their specific symptoms. These handouts described approaches for symptom management. For example, individuals with sleeping issues were provided with handouts describing available sound pillows and one that listed sleep hygiene tips. Other handouts included relaxation, and another covered concentration tips. Yet another handout listed website resources. These handouts were in addition to exercises performed during the counseling session itself, such as attention shifting or relaxation exercises. Both types of counseling were interactive, with constant feedback by the audiologist to encourage participant understanding and questioning. Critical to successful counseling was the interaction between audiologist and patient, notwithstanding the study protocols. However, as is typical of most randomized trials and with counseling approaches, no formal methods, such as testing, were used to ensure the participant’s understanding of the concepts presented during counseling. Role playing or pre-post testing could also have been used to assess receipt of the counseling concepts [3, 6, 7].

### Enactment

Enactment includes processes to monitor and improve the ability of each participant to implement the assigned intervention in real-life settings. Enactment is not the equivalent of treatment efficacy, because although a participant may have received the intervention as assigned, he or she may be unwilling or unable to act on that intervention [18]. Nevertheless, the efficacy of treatment can be viewed as a partial measure of enactment. In the TRTT, all participants were followed for 18 months following treatment onset. The impact of these intervention strategies in the TRTT was indirectly assessed by change in the Tinnitus Questionnaire, a tinnitus-specific health-related quality of life instrument [12]. Possible ways to measure enactment that could have been implemented include direct observation of learned skills, questionnaires or interviews of participants, or self-report [6].

## Results

Twenty-five audiologists were certified for TC and/or SOC counseling and 24 completed at least one counseling session.

Twenty-five audiologists submitted audiotapes for TC. Of these, 22 (88%) demonstrated competency for TC. An additional 2 (8%) audiologists demonstrated partial competency requiring a telephone call with the protocol monitor regarding some deficiencies in the recordings. One of these two audiologists re-submitted an audiotape demonstrating full competency. The other audiologist did not submit an acceptable audiotape and chose not to re-submit a second one.

Twenty-five audiologists submitted audiotapes for SOC. Of these, 15 (60%) demonstrated competency for SOC, with 2 audiologists demonstrating adequate competency but requiring a telephone call with the protocol monitor. The remaining 8 (32%) audiologists did not submit an acceptable audiotape, and 2 of these chose not to submit a second audiotape. Of 6 audiologists who submitted a second audiotape, 2 were classified as acceptable and 3 as partially acceptable. The remaining audiologist re-submitted another two audiotapes with the fourth classified as acceptable.

Audiologists submitted 101 checklists comprised of 33 items for TC (Table 3). Per the study protocol, although all sessions were recorded, only the first two treatment sessions of each type of counseling for each audiologist were reviewed by the protocol monitor for adherence to protocol. One randomly selected audiotape from among the next five counseling sessions also was reviewed. Of the 101 audiotapes and checklists submitted, protocol monitors reviewed 37 TC counseling sessions. Adherence to each of 33 critical items on the TC checklist as assessed by the protocol monitor ranged from 70 to 100% across 37 counseling sessions (median 97%). There was no difference between adherence for TRT (median, 97%) and Partial TRT (median, 100%) (data not shown). Only one checklist item, “Summary reviewed” (70% agreement), failed to achieve adherence of at least 80%.

**Table 3:** TC protocol adherence and comparison as assessed by counselor and comparison between counselor and monitor’s review of checklist

	All checklists	Checklists comparison	
Item	Audiologist (***n*** = 101)	Audiologist (***n*** = 37)	Protocol Monitor (***n*** = 37)
Data forms submitted	***N*** (%)	***N***	***N*** (%)
**A. Overview, goals**			
Overview of directive counseling and its goals described	101 (100)	37	35 (95)
Results of audiometric tests explained	101 (100)	37	37 (100)
Results of Loudness Discomfort Levels (LDL/UCL) explained	97 (96)	35	33 (89)
Results of tinnitus pitch match explained	98 (97)	36	36 (100)
Results of tinnitus loudness match explained	97 (96)	36	36 (100)
**B. Auditory function**			
Overview of auditory system described	101 (100)	37	37 (100)
Associate any sensorineural component to anatomical structure described	99 (98)	36	32 (89)
Physiology of hearing explained	101 (100)	37	37 (100)
Ear structure acts as a transformer—“hearing is perception at brain” explained	101 (100)	37	35 (95)
**C. Sensory system**			
Anatomy and function of outer and inner hair cells described	101 (100)	37	35 (95)
“Gain” of the auditory system explained	92 (91)	34	28 (82)
Cochlear structure explained (frequency-specific and constant nerve firing)	101 (100)	37	37 (100)
Function of the auditory nerve explained	101 (100)	37	32 (89)
Function of afferent and efferent nerve fibers explained	95 (94)	34	29 (85)
**D. Cortical and subcortical systems**			
Cortical areas explained	101 (100)	37	37 (100)
Subcortical areas (monitor, filter, and enhance) explained	101 (100)	37	37 (100)
Cortical functions (i.e., cognition) and sub-cortical functions (subconscious) described	99 (98)	37	37 (100)
Selective perception explained	100 (99)	37	36 (97)
Sensory contrast explained	100 (99)	37	35 (95)
Heller and Bergman study explained	100 (99)	37	37 (100)
Prioritization explained	100 (99)	37	33 (89)
Damage to OHCs and implications described	99 (98)	35	31 (89)
Sub-cortical monitoring of auditory input and neural patterns described	101 (100)	37	31 (84)
Classification of new or changed neural patterns explained	92 (91)	32	30 (94)
**E. Jastreboff neurophysiological model**			
Block diagram of Jastreboff model described	101 (100)	37	37 (100)
Cochlea as source of tinnitus described	101 (100)	37	36 (97)
Function of sub-cortical structures to filter random, unimportant sounds and detect new or different ones described	101 (100)	37	37 (100)
Relationship between emotional associations at the level of the limbic system and annoyance described	101 (100)	37	37 (100)
Activation of the autonomic nervous system causes the brain to prioritize tinnitus described	101 (100)	37	36 (97)
Activation of subconscious and conscious loops described	101 (100)	37	30 (81)
**D. Treatment goal and summary**			
First treatment goal: habituation of the reaction (annoyance to the tinnitus) discussed	101 (100)	37	36 (97)
Second treatment goal: habituation of the perception (awareness of the tinnitus) discussed	101 (100)	37	33 (89)
Summary reviewed	101 (100)	37	26 (70)

Audiologists submitted 45 checklists for SOC counseling sessions comprised of 44 items (Table 4). Of these, 30 audiotapes were reviewed by the protocol monitor. Adherence to each of 44 critical items on the SOC checklist across 30 SOC counseling sessions ranged from 42 to 100% (median, 87.5%). Some items failed to achieve adherence of at least 80%, including “Communicated empathy and understanding of Participant’s thought and feelings” (63%), “Emphasized rationale or/relevance of this particular recommendation” for stress (65%) or sleep issues (63%), “Changes Participant thinks would be helpful” for sleep (42%), “Importance of ability to concentrate” (58%), and various items under the topic of concentration (54–57%). Most of these topics are related to the patient-centered approach, suggesting that this approach was somewhat difficult for counselors to deliver, notwithstanding the previous training.

**Table 4:** SOC protocol adherence as assessed by counselor and comparison between counselor and monitor’s review of checklist

	All checklists	Checklist comparison	
Item checked by:	Audiologists (***n*** = 45)	Audiologists (***n*** = 30)	Protocol Monitor (***n*** = 30)
Data forms submitted	***N*** (%)	***N***	***N*** (%)
**A. Narrative topic**			
Study Participant narrative (“Tell me about your tinnitus”) elicited	45 (100)	30	30 (100)
Cognitive/affective key points in Participant’s narrative summarized	45 (100)	30	27 (90)
“Is there anything else you would like me to know about your tinnitus?” asked of Participant	45 (100)	30	24 (80)
“Do you worry about your tinnitus? What worries you?” asked of Participant	44 (98)	30	27 (90)
Key point about tinnitus “What it is & What it is not” reviewed	45 (100)	30	28 (93)
Key point about tinnitus “Noticing & Ignoring it” reviewed	44 (98)	30	27 (90)
Communicated empathy and understanding of Participant’s thoughts and feelings	45 (100)	30	19 (63)
**B. Hearing mechanism topic**			
Outer ear described	44 (98)	30	30 (100)
Middle ear described	44 (98)	30	30 (100)
Conductive hearing loss described	44 (98)	30	25 (83)
Inner ear, hair cells, cochlea and auditory nerve described	45 (100)	30	30 (100)
Vestibular system described	44 (98)	30	25 (83)
Sensorineural hearing loss described	44 (98)	30	27 (90)
**C. Audiometric/tinnitus/hyperacusis evaluation**			
Pure tone audiogram described	44 (98)	30	30 (100)
Speech tests described	38 (84)	30	26 (87)
Acoustic immittance described	44 (98)	30	25 (83)
Tinnitus pitch match described	44 (98)	30	27 (90)
Tinnitus loudness match described	42 (93)	30	26 (87)
**D. Coping with tinnitus and/or problem area topic**			
Main problem areas of Participant identified	45 (100)	30	28 (93)
Effective ways participant has coped with tinnitus in the past reinforced	45 (100)	30	24 (80)
Use of environmental sound described	45 (100)	30	28 (93)
Specific environmental sound devices described	45 (100)	30	28 (93)
**E. Stress topic**			
Stress discussed as a problem area	32 (71)	23	22 (96)
Stress reduction programs discussed	34 (76)	23	23 (100)
Relaxation exercises demonstrated	32 (71)	23	19 (83)
Emphasized rationale for/relevance of this particular recommendation in view of Participant’s specific complaint.	40 (89)	26	17 (65)
**F. Sleep issue topics**			
Sleep discussed as problem area	37 (82)	26	25 (96)
Healthy sleep patterns reviewed	38 (84)	26	26 (100)
Variables that interfere with sleep discussed	37 (82)	26	23 (88)
General recommendations for sleep environment described	37 (82)	26	26 (100)
Recommendations for sound therapy to enhance sleep described	38 (84)	26	26 (100)
Changes study participant thinks would be most helpful to minimize tinnitus interference with his/her sleep identified	36 (80)	26	11 (42)
Emphasized rationale for/relevance of this particular recommendation in view of Participant’s specific complaint	39 (87)	27	17 (63)
**G. Concentration issue topics**			
Concentration discussed as a problem area	34 (76)	26	23 (88)
Importance of ability to concentrate: memory, productivity, and job performance discussed	33 (73)	26	15 (58)
Use of environmental sounds to enhance concentration ability discussed	38 (84)	26	20 (77)
Attention shifting described	40 (89)	28	23 (82)
Shifting visual and auditory attention exercises conducted	35 (78)	28	16 (57)
Changes in work habits and environment, including short breaks, recommended	34 (76)	28	15 (54)
Tips for staying focused and engaged described	32 (71)	28	15 (54)
Emphasized rationale for/relevance of this particular recommendation in view of study Participant’s specific complaint	37 (82)	28	16 (57)
**H. Recommendations, summary, and treatment**			
Participant’s area(s) of concern summarized	45 (100)	30	24 (80)
Participant’s choices of treatment options for target areas discussed	45 (100)	30	24 (80)
Participant’s ability to cope with tinnitus (self-efficacy) reinforced	45 (100)	30	22 (73)

## Discussion

In the TRTT, adherence with the main components of both the TC and SOC counseling interventions was assessed by comparison of the checklists completed by the audiologist with the corresponding review by the protocol monitors. Rigorous review by the protocol monitors generally showed agreement with the audiologists’ perception. Audiologists’ perception of adherence and their self-report of adherence tended to be somewhat more favorably rated than that by the protocol monitor. Items that were less in agreement in the SOC counseling checklist tended to be those most related to the patient-oriented approach, which focuses on shared-decision making and goal setting [10]. Although the study audiologists routinely provided counseling of tinnitus patients in their clinical practices prior to the TRTT, most were not experienced with the SOC patient-oriented approach to managing tinnitus in the trial. This inexperience is reflected in the poorer performance during the initial competency assessment compared with their performance on TC counseling. Thus, the less favorable agreement of the patient-oriented SOC checklist items is not a surprising outcome. In retrospect, additional training seems to have been warranted for the SOC arm of the trial.

One challenge we faced while the trial was ongoing was the constant turnover in audiologists. This high turnover, primarily due to re-posting of the military study personnel participating in the trial, required constant training to ensure consistency and faithfulness to the interventions from the beginning to the end of the trial. In addition, the quality assurance procedures, including checklists and audiotape review, continued throughout the trial. Because this “extended time period” could impact treatment fidelity [14], monitoring procedures put into place continued to be maintained until trial end.

Treatment efficacy may indirectly reflect treatment fidelity. If so, then on average, all study groups in the TRTT may be judged to have been faithful to the intervention protocol. The majority of participants in all groups showed improvement on average by at least 30% in the Tinnitus Questionnaire at 18 months and improvement on all other tinnitus-specific health-related quality of life instruments used as secondary measures [11]. It is possible that a participant may have received the intervention, but chose not to or was unable to act upon it. Investigators who have examined this concept have found mixed results, i.e., some found a relation while others have not. However, it appears clear that there is a difference between treatment effectiveness and enactment and that enactment is not necessarily an element of treatment fidelity [8].

Although we are unable to explore fidelity further because the trial has ended, we would suggest that other investigators query both interventionists and participants following the intervention to understand their perspective on the treatment. For example, questions we might have asked the audiologists would be items such as preferences for one versus the other treatment arm or how likely they are to continue with either treatment arm in their practice. We would query study participants on how much they believe the treatment helped them and if they plan to continue using the skills they might have acquired during the trial.

### Challenges

Treatment fidelity encompasses both adherence and competency in the administration of a standard protocol. The goal of the TRTT was not only to develop measures to train audiologists in the practice of two distinct treatment interventions for tinnitus, but also to ensure that these interventions were delivered faithfully. Adherence to the treatment was absolutely necessary to ensure the findings of the trial were due to the intervention and not to other influences (e.g., counselor preference). The primary challenge we faced was that the same audiologist might be called upon to administer both interventions due to cost and personnel constraints. As noted by others, we needed to consider the nature of each intervention, the study design, and the relevant outcome measurements [7]. Our goal was to duplicate the interventions as currently in use in clinical practice, ensuring that each remained faithful to the original intervention.

Development of a standardized protocol for TRT was challenging for the TRTT. Typically, training in TRT had been offered through a 2 or 3-day course by Jastreboff, the TRT developer. His course also covered hyperacusis and misophonia, conditions that may accompany tinnitus. To our knowledge, Jastreboff used no specific measures to assess quality control by professionals within the course. For implementation in the TRTT, we had to operationalize and standardize the practice of TRT, which we modeled based on the delivery of TC and ST at the University of Maryland Tinnitus and Hyperacusis Center [19] and described in the literature [17]. To facilitate adherence and prevent drift across the timespan of the trial, we developed integrated scripts, checklists, and visual aids. In addition, we required competency assessment of audiologists before providing treatment, including review of TRT audiotapes.

Development of the protocol for SOC was even more of a challenge than that for TC. In contrast to the structured TC protocol, SOC engaged the participant and was more fluid from individual to individual, a known barrier to implementing treatment fidelity with patient-focused interventions [20]. It was necessary to determine how much flexibility was allowable within the confines of the SOC protocol to ensure acceptable implementation of the individualized treatment plan while still allowing for collaborative goal-setting, which was critical for treatment efficacy with SOC. To implement the treatment fidelity portion of SOC, we developed scripts with the option to skip over sections that were not of importance to the individual, and with more focus on those issues considered critical by him or her [10]. We ruled out the use of a flipchart for SOC, which would have been too circumscribed for the patient-centered approach. Instead, we relied more on handouts describing ways to deal with specific symptoms, with only those important to the study participant reviewed and distributed to the individual.

Another challenge was training the audiologists. For some audiologists, TRT and SOC provided new concepts in tinnitus care. Most audiologists are used to taking the lead in delivering care for tinnitus, so it was difficult for some to move from the usual clinician-patient interaction in a position of authority towards collaboration decision-making and elicitation of the patient’s personal story while administering SOC. Associated with the SOC approach was the need to show empathy, which was challenging for some audiologists. This difficulty may be reflected in the lack of agreement between the audiologist and protocol monitor on items related to the patient-centered approach on the SOC checklist. For both interventions, audiologists needed to learn how to use scripts effectively—not reading them verbatim, but placing the critical concepts in their own words while still engaging with the study patient. Most audiologists were able to present the counseling content effectively in the case of TC because they were aided by the talking points on the back of the flip-chart.

### Limitations

The implementation of treatment fidelity in the TRTT had some limitations. We had only a single protocol monitor for each type of counseling. Most experts recommend that two monitors should be involved and inter-rater scoring completed. With only one protocol monitor, there could be some bias in selection of the critical components to include in the protocol and review of the audiotapes, leading to some error in absolute measurement of adherence. However, there was high agreement between audiologists and protocol monitors when scoring the checklists. Agreement in scores between 80 and 100% typically indicates high integrity of the intervention with the protocol [18]. Another possible limitation is a protocol preference voiced by some audiologists or the possibility that some participants would have fared better with one type of counseling versus another. Although these audiologist and participant characteristics may have impacted treatment integrity and possibly outcomes, the randomized study design should have mitigated these effects when comparing the efficacy of the two interventions. A third limitation is that we did not directly measure engagement. That is, we did not collect any information from study participants about whether they understood or were engaged in the counseling. We did, however, measure the time that each counseling session took, finding that audiologists spent sufficient and similar time with participants to have covered the critical components. Although we did not ask study participants about the acceptability of the treatment, there was shared goal setting in TC and shared decision making with SOC [9, 10]. Study participant input was essential for both interventions in the TRTT, and these activities required some level of enactment.

### Strengths

One major strength of the TRTT treatment fidelity measures was the development of standardized protocols that can readily be adapted to clinical settings for either type of counseling. By describing the steps required to implement each of the two types of counseling in detail, other audiologists or clinicians can provide these types of treatments for individuals with tinnitus. In fact, the protocols developed by the TRTT are currently in use in some of the clinics participating in the TRTT. The high turnover of audiologists and constant training had the unintended effect of resulting in a large number of audiologists trained in these two interventions. The viewpoint expressed anecdotally by participating audiologists was that the TRTT and associated treatment fidelity steps served to strengthen their professional skills, which we used originally as a selling point to promote the trial to the military commands at each site in the TRTT.

In conclusion, the TRTT ensured treatment fidelity to two types of counseling interventions, TC and SOC, for the management of severe tinnitus, following the guidelines of the NIH Behavior Change Consortium [1]. Although there was no appreciable difference between treatment groups in terms of efficacy, we believe this lack of difference cannot be attributed to lack of implementation and differentiation of the interventions. The effort required to design treatment fidelity strategies required careful consideration and thoughtful planning to integrate these strategies into the clinical setting. The reward for this thought and effort is that the treatment protocols are replicable and that the trial results can be trusted.

## Supplementary information

**Additional file 1.**

## Data Availability

The datasets used and/or analyzed during the current study are available from the corresponding author on reasonable request.

## Abbreviations

ASHA: American speech-Language-Hearing Association
NIH: National Institutes of Health
SG: Sound generator
SOC: Standard of care
ST: Sound therapy
TC: Tinnitus-specific educational counseling aspect of tinnitus retraining therapy
TQ: Tinnitus Questionnaire
TRT: Tinnitus retraining therapy
TRTT: Tinnitus Retraining Therapy Trial
