# Supplementary material for: Treatment fidelity in the Tinnitus Retraining Therapy Trial
Source: Trials. 2020 Jul 23;21:670. doi: 10.1186/s13063-020-04530-9 (PMC7376906; doi:10.1186/s13063-020-04530-9)
Supplement: Supplementary file 1 — Additional file 1. [file 13063_2020_4530_MOESM1_ESM.pdf]

Supplemental files

1. **Certification requirements for audiologists in the TRTT.**

2. **TC Checklist.** This form is the checklist used by audiologist during a TRT intervention session and covers both TC counseling and SG fitting

3. **SOC Checklist**. This form is the checklist used by audiologist during a SOC intervention session

4. **TC Review Checklist**. This form is the checklist used by the protocol monitor to evaluate the TC counseling session

5. **SOC Review Checklist**. This form is the checklist used by the protocol monitor to evaluate the SOC counseling session

Table 1. Certification requirements for audiologists in the TRTT.

Certification of audiologists to participate in the TRTT required the following:

- Demonstrate completion of one or more years of clinical experience
- Attend general, TRT, and SOC training meetings
- Read relevant sections of the MOP
- Review data collection forms related to intervention and audiologic measurements
- Review of TC, SOC, and ST videotapes
- Complete online TRTT Knowledge assessment test
- Complete two sets of baseline eligibility and randomization forms
- Submit one voice recording of TC and one of SOC session

2. TC Checklist

Reference No.: ___ ___ ___ ___ ___ ___

Tinnitus Retraining Therapy Trial
TRT Checklist

Purpose: Records topics and information covered during the Directive Counseling and sound generator fitting sessions for Study Participants assigned to tinnitus retraining therapy (TRT) in the TRTT
When: At the Initial Treatment Visit (T1)
Completed by: Study Audiologist who completed the Directive Counseling and sound generator fitting, and Clinical Coordinator
Information obtained from: Study Audiologist
Instructions: This form is completed for each Study Participant assigned to TRT. Indicate whether each of the indicated tasks were completed or topic areas covered during the counseling session by placing a check mark (✔) or (**X**) in the box headed by a "No" or "Yes." Please enter this form on the online TRTT database within one week of completion of the visit and file the paper copy in the Study Participant's TRTT file at the Clinical Center.

A. Clinic, Patient, and Visit Identification

1. Clinic ID: ___ ___ ___ ___
2. Patient ID: T ___ ___ ___ ___
3. Patient four-letter code: ___ ___ ___ ___
4. Date form completed: ___ ___ - ___ ___ ___ - ___ ___ ___ ___ (day, mo, year)
5. Visit ID: T 1
6. Form revision date: 2 1 - J U L - 2 0 1 1 (day, mo, year)

Task or Topic

	**B. Preparation**	**No**	**Yes**
7.	Visual aids (three-dimensional model of the ear and flip-chart) set up	( )$_0$	( )$_1$
8.	Voice recorder set up and checked	( )$_0$	( )$_1$
9.	Copies of the Study Participant's audiological/tinnitus/hyperacusis examination results prepared	( )$_0$	( )$_1$

	**C. Directive Counseling Session**	**No**	**Yes**
10.	Overview of directive counseling and its goals described	( )$_0$	( )$_1$
11.	Results of audiometric tests explained	( )$_0$	( )$_1$
12.	Results of Loudness Discomfort Levels (LDL/UCL) explained	( )$_0$	( )$_1$
13.	Results of tinnitus pitch match explained	( )$_0$	( )$_1$
14.	Results of tinnitus loudness match explained	( )$_0$	( )$_1$

	Task or Topic	No	Yes
15.	Overview of auditory system described	( )0	( )1
16.	Associate any sensorineural component to anatomical structure described	( )0	( )1
17.	Physiology of hearing explained	( )0	( )1
18.	Ear structure acts as a transformer - 'hearing is perception at brain' explained	( )0	( )1
19.	Anatomy and function of outer and inner hair cells described	( )0	( )1
20.	"Gain" of the auditory system explained	( )0	( )1
21.	Cochlear structure explained (frequency- specific and constant nerve firing)	( )0	( )1
22.	Function of the auditory nerve explained	( )0	( )1
23.	Function of afferent and efferent nerve fibers explained	( )0	( )1
24.	Cortical areas explained	( )0	( )1
25.	Subcortical areas (monitor, filter, and enhance) explained	( )0	( )1
26.	Cortical functions (i.e., cognition) and sub-cortical functions (subconscious) described	( )0	( )1
27.	Selective perception explained	( )0	( )1
28.	Sensory contrast explained	( )0	( )1
29.	Heller and Bergman study explained	( )0	( )1
30.	Prioritization explained	( )0	( )1
31.	Damage to OHCs and implications described	( )0	( )1
32.	Sub-cortical monitoring of auditory input and neural patterns described	( )0	( )1
33.	Classification of new or changed neural patterns explained	( )0	( )1
34.	Block diagram of Jastreboff model described	( )0	( )1
35.	Cochlea as *source* of tinnitus described	( )0	( )1
36.	Function of sub-cortical structures to filter random, unimportant sounds and detect new or different ones described	( )0	( )1
37.	Relationship between *emotional associations* at the level of the limbic system and *annoyance* described	( )0	( )1
38.	Activation of the autonomic nervous system causes the brain to *prioritize* tinnitus described	( )0	( )1
39.	Activation of subconscious and conscious loops described	( )0	( )1
40.	First treatment goal: habituation of the reaction (annoyance to the tinnitus) discussed	( )0	( )1
41.	Second treatment goal: habituation of the perception (awareness of the tinnitus) discussed	( )0	( )1
42.	Summary reviewed	( )0	( )1
43.	Copy of the summary given to Study Participant	( )0	( )1
44.	Session recorded	( )0	( )1

45. Length of counseling session:
    ( )$_0$ Less than 30 minutes
    ( )$_1$ 30 minutes or more but less than 1 hour
    ( )$_2$ 1 hour or more but less than 90 minutes
    ( )$_3$ 90 minutes or more but less than 2 hours
    ( )$_4$ 2 or more hours

D.	**Sound Generator Fitting**	**No**	**Yes**
46.	Copy of the "Guidelines for Instrument Use" given to Study Participant..................	( )$_0$	( )$_1$
47.	Instrument use and volume settings described...	( )$_0$	( )$_1$
48.	"Set it and forget it" explained..	( )$_0$	( )$_1$
49.	"Avoiding silence" discussed...	( )$_0$	( )$_1$
50.	"Keep the focus off the tinnitus" discussed...	( )$_0$	( )$_1$
51.	Six-week effect explained..	( )$_0$	( )$_1$
52.	Rough patches explained..	( )$_0$	( )$_1$
53.	Goals reviewed..	( )$_0$	( )$_1$
54.	Follow-up procedures and schedule explained to Study Participant........................	( )$_0$	( )$_1$

E. Administrative Information

55. Date form reviewed by Study Audiologist: ___ ___ - ___ ___ ___- ___ ___ ___ ___
    day mo year

56. Study Audiologist ID: ___ ___ ___

57. Study Audiologist signature: ______________________________

58. Date form reviewed by Clinical Coordinator: ___ ___ - ___ ___ ___- ___ ___ ___ ___
    day mo year

59. Clinical Coordinator ID: ___ ___ ___

60. Clinical Coordinator signature: ______________________________

3. SOC Checklist

Reference No.: ___ ___ ___ ___ ___ ___

Tinnitus Retraining Therapy Trial
Standard of Care Checklist

Purpose: Records topics and information covered during a Standard of Care (SC) counseling session in the TRTT for Study Participants assigned to SC
When: At the Initial Treatment Visit (T1)
Completed by: Study Audiologist who completed the SC counseling session and the Clinical Coordinator
Information obtained from: Study Audiologist
Instructions: This form is completed for each Study Participant assigned to Standard of Care. Record the kit number issued at randomization and indicate whether the following tasks were completed or topic areas covered during the counseling session by placing a check mark (✔) or (**X**) in the box headed by a "No" or "Yes". Please enter this form on the online TRTT database within one week of completion of the visit in which the SC counseling took place and file the paper copy in the Study Participant's TRTT file at the Clinical Center.

A. Clinic, Patient, and Visit Identification

1. Clinic ID: ___ ___ ___ ___
2. Patient ID: T ___ ___ ___ ___
3. Patient four-letter code: ___ ___ ___ ___
4. Date form completed: ___ ___ - ___ ___ ___ - ___ ___ ___ ___ (day mo year)

   NOTE: This date must be the same date that the T1 visit took place.
5. Visit ID: T 1
6. Form revision date: 2 3 - J A N - 2 0 1 2 (day mo year)

B. Kit Number

7. Indicate the kit number of the empty sound generator kit issued at randomization. Write the Patient ID and four-letter code on the tear-off label from the empty sound generator kit and affix the tear-off label here:

   Write the Patient ID and four-letter code on the tear-off label and affix here

   7a. Enter kit number online

C.	Preparation	No	Yes
8.	Three-dimensional model of the ear set up	( )0	( )1
9.	Handouts available	( )0	( )1
10.	Voice recorder set up and checked	( )0	( )1
11.	Copies of the Study Participant's audiological/tinnitus/hyperacusis examination results prepared	( )0	( )1

			No	Yes
**D.**		**Narrative**	**No**	**Yes**
	12.	Study Participant narrative (“Tell me about your tinnitus”) elicited	( )$_0$	( )$_1$
	13.	Cognitive/affective key points in Study Participant’s narrative summarized	( )$_0$	( )$_1$
	14.	“Is there anything else you would like me to know about your tinnitus?” asked of Study Participant	( )$_0$	( )$_1$
	15.	“Do you worry about your tinnitus? What worries you?” asked of Study Participant	( )$_0$	( )$_1$
	16.	Key point about tinnitus “What it is & What it isn’t” reviewed	( )$_0$	( )$_1$
	17.	Key point about tinnitus “Noticing & Ignoring it” reviewed	( )$_0$	( )$_1$
	18.	Copy of the handout “Key points about tinnitus” given to Study Participant	( )$_0$	( )$_1$
	19.	Communicated empathy and understanding of Study Participant's thoughts and feelings	( )$_0$	( )$_1$
**E.**		**Hearing Mechanism**	**No**	**Yes**
	20.	Outer ear described	( )$_0$	( )$_1$
	21.	Middle ear described	( )$_0$	( )$_1$
	22.	Conductive hearing loss described	( )$_0$	( )$_1$
	23.	Inner ear, hair cells, cochlea and auditory nerve described	( )$_0$	( )$_1$
	24.	Vestibular system described	( )$_0$	( )$_1$
	25.	Sensorineural hearing loss described	( )$_0$	( )$_1$
**F.**		**ATH Evaluation**	**No**	**Yes**
	26.	Pure tone audiogram described	( )$_0$	( )$_1$
	27.	Speech tests described	( )$_0$	( )$_1$
	28.	Acoustic immittance described	( )$_0$	( )$_1$
	29.	Tinnitus pitch match described	( )$_0$	( )$_1$
	30.	Tinnitus loudness match described	( )$_0$	( )$_1$
**G.**		**Coping with Tinnitus and/or Problem Areas**	**No**	**Yes**
	31.	Main problem areas of Study Participant identified	( )$_0$	( )$_1$
	32.	Effective ways Study Participant has coped with tinnitus in the past reinforced	( )$_0$	( )$_1$
	33.	Use of environmental sound described	( )$_0$	( )$_1$
	34.	Specific environmental sound devices described	( )$_0$	( )$_1$
	35.	Copy of handout ”Environmental Sound Devices” given to Study Participant	( )$_0$	( )$_1$
**H.**		**Stress**	**No**	**Yes**
	36.	Was stress discussed as a problem area?	( )$_0$	( )$_1$
	37.	Stress reduction programs discussed	( )$_0$	( )$_1$
	38.	Relaxation exercises demonstrated	( )$_0$	( )$_1$
	39.	Handout “Relaxation Tips” from www.helpguide.org given to Study Participant	( )$_0$	( )$_1$
	40.	Emphasized rationale for/relevance of this particular recommendation in view of Study Participant's specific complaint	( )$_0$	( )$_1$

		No	Yes
**I.**	**Sleep Issues**	**No**	**Yes**
41.	Was sleep discussed as a problem area?	($)_0$	($)_1$
42.	Healthy sleep patterns reviewed	($)_0$	($)_1$
43.	Variables that interfere with sleep discussed	($)_0$	($)_1$
44.	General recommendations for sleep environment described	($)_0$	($)_1$
45.	Recommendations for sound therapy to enhance sleep described	($)_0$	($)_1$
46.	Changes Study Participant thinks would be most helpful to minimize tinnitus interference with his/her sleep identified	($)_0$	($)_1$
47.	Handout “Sleep tips from the Mayo Clinic” given to Study Participant	($)_0$	($)_1$
48.	Emphasized rationale for/relevance of this particular recommendation in view of Study Participant's specific complaint	($)_0$	($)_1$
**J.**	**Concentration**	**No**	**Yes**
49.	Was concentration discussed as a problem area?	($)_0$	($)_1$
50.	Importance of ability to concentrate: memory, productivity, and job performance discussed	($)_0$	($)_1$
51.	Use of environmental sounds to enhance concentration ability discussed	($)_0$	($)_1$
52.	Handout “Concentration tips from the University of Cambridge” given to Study Participant	($)_0$	($)_1$
53.	Attention shifting described	($)_0$	($)_1$
54.	Shifting visual and auditory attention exercises conducted	($)_0$	($)_1$
55.	Changes in work habits and environment, including short breaks, recommended	($)_0$	($)_1$
56.	Tips for staying focused and engaged described	($)_0$	($)_1$
57.	Emphasized rationale for/relevance of this particular recommendation in view of Study Participant's specific complaint	($)_0$	($)_1$
**K.**	**Recommendations, Summary, and Treatment**	**No**	**Yes**
58.	Study Participant’s area(s) of concern summarized	($)_0$	($)_1$
59.	Study Participant’s choices of treatment options for target areas discussed	($)_0$	($)_1$
60.	Study Participant’s ability to cope with tinnitus (self-efficacy) reinforced	($)_0$	($)_1$
61.	Study Participant advised about TRTT Follow-up visits and evaluations	($)_0$	($)_1$

62. Length of counseling session:
($)_0$ Less than 30 minutes
($)_1$ 30 minutes or more but less than 1 hour
($)_2$ 1 hour or more but less than 90 minutes
($)_3$ 90 minutes or more but less than 2 hours
($)_4$ 2 or more hours

L. Administrative Information

63. Date form reviewed by Study Audiologist: ___ ___ - ___ ___ ___- ___ ___ ___ ___
day mo year

64. Study Audiologist ID: ___ ___ ___

65. Study Audiologist signature: ______________________________

66. Date form reviewed by Clinical Coordinator: ___ ___ - ___ ___ ___- ___ ___ ___ ___
day mo year

67. Clinical Coordinator ID: ___ ___ ___

68. Clinical Coordinator signature: ______________________________

4. TC Review checklist

Reference No.: ___ ___ ___ ___ ___ ___

Tinnitus Retraining Therapy Trial
Review of Directive Counseling Form

Purpose: Records whether required topics were discussed during Directive Counseling sessions
When: Following submission of voice recordings for certification or adherence to treatment protocol
Completed by: TRT Protocol Monitor
Information obtained from: Voice Recording
Instructions: This form is completed by reviewing the voice recording of a Directive Counseling session submitted by a Study Audiologist for certification or for assessment of adherence to the treatment protocol. Indicate whether each of the indicated tasks were completed or topic areas discussed during the counseling session by placing a check mark (✔) or (**X**) in the box headed by a "No"or "Yes." Email or fax completed form to the Data Coordinating Center at fax (443) 451-8299 or dcc@trtt.org, and telephone (443) 287-6420 to confirm receipt of fax/email.

A. Clinic, Patient, and Visit Identification

Write N/A for Patient ID, Patient four-letter code, Visit ID, and Visit date if voice recording submitted for certification

1. Clinic ID: ___ ___ ___ ___
2. Patient ID: T ___ ___ ___ ___
3. Patient four-letter code: ___ ___ ___ ___
4. Date form completed: ___ ___ - ___ ___ ___ - ___ ___ ___ ___
   day mo year
5. Visit ID: ___ ___
6. Form revision date: 2 1 - J U L - 2 0 1 1
   day mo year
7. Visit date: ___ ___ - ___ ___ ___ - ___ ___ ___ ___
   day mo year

B. Study Audiologist Identification

8. Study Audiologist name: ______________________________
9. Study Audiologist ID: ___ ___ ___
10. Voice recording submitted for:
    ( )$_0$ Certification
    ( )$_1$ Re-submission for certification
    ( )$_2$ Assessing adherence to treatment protocol

C. Directive Counseling Session

	Task or Topic	No	Yes
11.	Overview of directive counseling and its goals described	( )$_0$	( )$_1$
12.	Results of audiometric tests explained	( )$_0$	( )$_1$
13.	Results of Loudness Discomfort Levels (LDL/UCL) explained	( )$_0$	( )$_1$
14.	Results of tinnitus pitch match explained	( )$_0$	( )$_1$
15.	Results of tinnitus loudness match explained	( )$_0$	( )$_1$

	Task or Topic	No	Yes
16.	Overview of auditory system described	( )0	( )1
17.	Association of any sensorineural component to anatomical structure described	( )0	( )1
18.	Physiology of hearing explained	( )0	( )1
19.	Ear structure acts as a transformer - 'hearing is perception at brain' explained	( )0	( )1
20.	Anatomy and function of outer and inner hair cells described	( )0	( )1
21.	"Gain" of the auditory system explained	( )0	( )1
22.	Cochlear structure explained (frequency- specific and constant nerve firing)	( )0	( )1
23.	Function of the auditory nerve explained	( )0	( )1
24.	Function of afferent and efferent nerve fibers explained	( )0	( )1
25.	Cortical areas explained	( )0	( )1
26.	Subcortical areas (monitor, filter, and enhance) explained	( )0	( )1
27.	Cortical functions (i.e., cognition) and sub-cortical functions (subconscious) described	( )0	( )1
28.	Selective perception explained	( )0	( )1
29.	Sensory contrast explained	( )0	( )1
30.	Heller and Bergman study explained	( )0	( )1
31.	Prioritization explained	( )0	( )1
32.	Damage to OHCs and implications described	( )0	( )1
33.	Sub-cortical monitoring of auditory input and neural patterns described	( )0	( )1
34.	Classification of new or changed neural patterns explained	( )0	( )1
35.	Block diagram of Jastreboff model described	( )0	( )1
36.	Cochlea as *source* of tinnitus described	( )0	( )1
37.	Function of sub-cortical structures to filter random, unimportant sounds and detect new or different ones described	( )0	( )1
38.	Relationship between *emotional associations* at the level of the limbic system and *annoyance* described	( )0	( )1
39.	Activation of the autonomic nervous system causes the brain to *prioritize* tinnitus described	( )0	( )1
40.	Activation of subconscious and conscious loops described	( )0	( )1
41.	First treatment goal: habituation of the reaction (annoyance to the tinnitus) discussed	( )0	( )1
42.	Second treatment goal: habituation of the perception (awareness of the tinnitus) discussed	( )0	( )1
43.	Summary reviewed	( )0	( )1

D. Assessment

44. Overall, the Directive Counseling session was:
    - ( )$_0$ Acceptable, **SKIP to item 48, Section E**
    - ( )$_1$ Acceptable, but needs improvement
    - ( )$_2$ Not acceptable

45. If needs improvement or not acceptable, indicate areas requiring remediation: ______________________

____

____

____

____

____

46. Is further training required?
    - ( )$_0$ No, **SKIP to item 48, Section E**
    - ( )$_1$ Yes

Indicate how further training will occur (check ‘Yes’ or ‘No’ to each item):

		**No**	**Yes**
47a.	Telephone call to the Study Audiologist.	( )$_0$	( )$_1$
47b.	Request to view videotape	( )$_0$	( )$_1$
47c.	Request to read Chapters 11 and 12 of the TRTT Manual of Procedures.	( )$_0$	( )$_1$
47d.	In-person training by Skype™..	( )$_0$	( )$_1$
47e.	In-person training at Clinical Center.	( )$_0$	( )$_1$
47f.	Other, specify: ______________________________	( )$_0$	( )$_1$

E. Administrative Information

48. Protocol Monitor ID: __ __ __

49. Protocol Monitor signature: ______________________________

5. SOC Review Checklist

Reference No.: ___ ___ ___ ___ ___ ___

Tinnitus Retraining Therapy Trial
Review of Standard of Care Form

Purpose: Records whether required topics were discussed during the Standard of Care (SC) Counseling sessions
When: Following submission of voice recordings for certification or adherence to treatment protocol
Completed by: SC Protocol Monitor
Information obtained from: Voice Recording
Instructions: This form is completed by reviewing the voice recording of a Standard of Care Counseling session submitted by a Study Audiologist for certification or for assessment of adherence to the treatment protocol. Indicate whether each of the indicated tasks were completed or topic areas discussed during the counseling session by placing a check mark (✔) or (**X**) in the box headed by a "No"or "Yes." Email or fax completed form to the Data Coordinating Center at fax (443) 451-8299 or dcc@trtt.org, and telephone (443) 287-6420 to confirm receipt of fax/email.

A. Clinic, Patient, and Visit Identification

Write N/A for Patient ID, Patient four-letter code, Visit ID, and Visit date if voice recording submitted for certification.

1. Clinic ID: ___ ___ ___ ___
2. Patient ID: T ___ ___ ___ ___
3. Patient four-letter code: ___ ___ ___ ___
4. Date form completed: ___ ___ - ___ ___ ___ - ___ ___ ___ ___ (day, mo, year)
5. Visit ID: ___ ___
6. Form revision date: 2 3 - J A N - 2 0 1 2 (day, mo, year)
7. Visit date: ___ ___ - ___ ___ ___ - ___ ___ ___ ___ (day, mo, year)

B. Study Audiologist Identification

8. Study Audiologist name: ______________________________
9. Study Audiologist ID: ___ ___ ___
10. Voice recording submitted for:
    ( )$_0$ Certification
    ( )$_1$ Re-submission for certification
    ( )$_2$ Assessing adherence to treatment protocol

Task or Topic

C.	Narrative	No	Yes
11.	Study Participant narrative ("Tell me about your tinnitus") elicited	( )$_0$	( )$_1$
12.	Cognitive/affective key points in Study Participant's narrative summarized	( )$_0$	( )$_1$
13.	"Is there anything else you would like me to know about your tinnitus?" asked of Study Participant	( )$_0$	( )$_1$
14.	"Do you worry about your tinnitus? What worries you?" asked of Study Participant	( )$_0$	( )$_1$

	Task or Topic	No	Yes
15.	Key point about tinnitus “What it is & What it isn’t” reviewed	( )$_0$	( )$_1$
16.	Key point about tinnitus “Noticing & Ignoring it” reviewed	( )$_0$	( )$_1$
17.	Communicated empathy and understanding of Study Participant's thoughts and feelings	( )$_0$	( )$_1$

D.	Hearing Mechanism	No	Yes
18.	Outer ear described	( )$_0$	( )$_1$
19.	Middle ear described	( )$_0$	( )$_1$
20.	Conductive hearing loss described	( )$_0$	( )$_1$
21.	Inner ear, hair cells, cochlea and auditory nerve described	( )$_0$	( )$_1$
22.	Vestibular system described	( )$_0$	( )$_1$
23.	Sensorineural hearing loss described	( )$_0$	( )$_1$

E.	ATH Evaluation	No	Yes
24.	Pure tone audiogram described	( )$_0$	( )$_1$
25.	Speech tests described	( )$_0$	( )$_1$
26.	Acoustic immittance described	( )$_0$	( )$_1$
27.	Tinnitus pitch match described	( )$_0$	( )$_1$
28.	Tinnitus loudness match described	( )$_0$	( )$_1$

F.	Coping with Tinnitus and/or Problem Areas	No	Yes
29.	Main problem areas of Study Participant identified	( )$_0$	( )$_1$
30.	Effective ways Study Participant has coped with tinnitus in the past reinforced	( )$_0$	( )$_1$
31.	Use of environmental sound described	( )$_0$	( )$_1$
32.	Specific environmental sound devices described	( )$_0$	( )$_1$

G.	Stress	No	Yes
33.	Was stress discussed as a problem area?	( )$_0$	( )$_1$
34.	Stress reduction programs discussed	( )$_0$	( )$_1$
35.	Relaxation exercises demonstrated	( )$_0$	( )$_1$
36.	Emphasized rationale for/relevance of this particular recommendation in view of Study Participant's specific complaint	( )$_0$	( )$_1$

H.	Sleep Issues	No	Yes
37.	Was sleep discussed as a problem area?	( )$_0$	( )$_1$
38.	Healthy sleep patterns reviewed	( )$_0$	( )$_1$
39.	Variables that interfere with sleep discussed	( )$_0$	( )$_1$
40.	General recommendations for sleep environment described	( )$_0$	( )$_1$
41.	Recommendations for sound therapy to enhance sleep described	( )$_0$	( )$_1$
42.	Changes Study Participant thinks would be most helpful to minimize tinnitus interference with his/her sleep identified	( )$_0$	( )$_1$
43.	Emphasized rationale for/relevance of this particular recommendation in view of Study Participant's specific complaint	( )$_0$	( )$_1$

I.	Concentration	No	Yes
44.	Was concentration discussed as a problem area?	( )$_0$	( )$_1$
45.	Importance of ability to concentrate: memory, productivity, and job performance discussed.	( )$_0$	( )$_1$
46.	Use of environmental sounds to enhance concentration ability discussed	( )$_0$	( )$_1$
47.	Attention shifting described	( )$_0$	( )$_1$
48.	Shifting visual and auditory attention exercises conducted	( )$_0$	( )$_1$
49.	Changes in work habits and environment, including short breaks, recommended	( )$_0$	( )$_1$
50.	Tips for staying focused and engaged described	( )$_0$	( )$_1$
51.	Emphasized rationale for/relevance of this particular recommendation in view of Study Participant's specific complaint	( )$_0$	( )$_1$

J.	Recommendations, Summary, and Treatment	No	Yes
52.	Study Participant's area(s) of concern summarized	( )$_0$	( )$_1$
53.	Study Participant's choices of treatment options for target areas discussed	( )$_0$	( )$_1$
54.	Study Participant's ability to cope with tinnitus (self-efficacy) reinforced	( )$_0$	( )$_1$

K. Assessment

55. Comments: ______________________________

____

____

____

____

____

56. Overall, the Standard of Care Counseling session was:
( )$_0$ Acceptable, **SKIP to item 60, Section L**
( )$_1$ Acceptable, but needs improvement
( )$_2$ Not acceptable

57. If needs improvement or not acceptable, indicate areas requiring remediation: ______________________________

____

____

____

____

____

58. Is further training required?
    ( )$_0$ No, **SKIP to item 60, Section L**
    ( )$_1$ Yes

Indicate how further training will occur (check 'Yes' or 'No' for each item):

		No	Yes
59a.	Telephone call to the Study Audiologist.	( )$_0$	( )$_1$
59b.	Request to view videotape.	( )$_0$	( )$_1$
59c.	Request to read Chapter 13 of the TRTT Manual of Procedures.	( )$_0$	( )$_1$
59d.	In-person training by Skype™..	( )$_0$	( )$_1$
59e.	In-person training at Clinical Center.	( )$_0$	( )$_1$
59f.	Other, specify: ______________________________	( )$_0$	( )$_1$

L. Administrative Information

60. Protocol Monitor ID: __ __ __

61. Protocol Monitor signature: ______________________________________
